# A preliminary investigation examining patient reported outcome measures for low back pain and utilisation amongst chiropractors in Australia: facilitators and barriers to clinical implementation

**DOI:** 10.1186/s12998-018-0208-9

**Published:** 2018-10-09

**Authors:** Natalie Clohesy, Anthony Schneiders

**Affiliations:** Department of Exercise & Health Sciences. School of Health, Medical and Applied Sciences, Central Queensland University, University Dr, Branyan, QLD 4670 Australia

**Keywords:** Patient reported outcome measures, Low back pain, Chiropractic

## Abstract

**Background:**

The current utilisation of patient reported outcome measures (PROMs) for low back pain (LBP) within the Australian Chiropractic profession is unknown. The aims of this study were to determine the current utilisation of LBP PROMs amongst Chiropractors in Australia and to identify the potential barriers and facilitators of using PROMs for LBP in Chiropractic practice.

**Methods:**

A cross sectional online survey was distributed to Chiropractors in Australia who were members of the Chiropractic Association of Australia (CAA) and Chiropractic Australia (CA) between June–August 2016. Three thousand fourteen CAA members and 930 CA members were invited to participate totaling 3944 potential participants.

**Results:**

The findings from this survey provides baseline data for the prevalence of LBP PROMs within the Australian Chiropractic profession. A total of 558 participants completed the survey reflecting a response rate of 14.1%. 72.5% of respondents used LBP PROMs in clinical practice. PROMs were categorised into pain, function and health. At initial patient consultations the most commonly used pain PROMs were the pain diagram, Visual Analogue Scale and Numeric Rating Scale. Most commonly used functional LBP PROMs were the Oswestry Disability Index, Functional Rating Index and Roland Morris Questionnaire. The Health Status Questionnaire (HSQ) was the most commonly used health LBP PROM followed by RAND Health Questionnaires.

**Conclusion:**

Most of the survey respondents use PROMs in clinical practice. The most common barrier chiropractors identified that prevent LBP PROM utilisation was the lack of operational definition surrounding PROMs, as well as how to use them and the perception that they are time consuming. Facilitatory factors to implement PROMs included using simple administration systems, utilising electronic forms and consistent implementation. This research indicates that there is a potential need to further educate the Chiropractic profession regarding PROMs.

## Background

Low back pain (LBP) is a major health issue globally [1] due to its high prevalence and financial cost to the individual, private health funds and the public health care systems [2]. It is estimated that in Australia 70–90% of people will suffer from LBP at some point in their lives [1]. According to the Australian Bureau of Statistics (ABS) national health survey, a staggering 3 million Australians (14% of the population), suffer from LBP annually [3]. Patients suffering from LBP seek treatment from a range of different health care providers including chiropractors.

Providing the most appropriate and efficacious treatment for patients with LBP decreases the financial burden on the patient and the health care system [4]. To accurately document treatment benefits and potential risks, patient reported outcome measures (PROMs) can be utilised [5]. When used appropriately, PROMs can measure the change in health status, or the lack thereof, in three critical areas; pain management, physical impairment and disability [6].

Selecting and applying PROMs correctly when treating patients with LBP enables chiropractors to provide evidence of treatment outcomes. Recent evaluation of the literature has demonstrated that PROMs are used within Chiropractic research [7]. However, the current utilisation of PROMs for LBP among chiropractors in clinical settings in Australia remains unknown.

While the widespread adoption of PROMs by chiropractors is uncertain in Australia, there is some data on chiropractors in other countries and other allied health providers. A survey of chiropractors in North America was completed by Hinton et al., [8]. A postal survey of all registrants of the Chiropractors’ Association of Saskatchewan resulted in a response rate of 38% with the findings suggesting that the majority of chiropractors do not use psychosocial questionnaires or condition-specific disability instruments to document patient’s changes in health status.

Chiropractors and physiotherapists are trained in the diagnosis and management of musculoskeletal conditions, and there is considerable overlap in the types of conditions that are encountered clinically [9]. Abrams et al., [9] was the first to report on the use of standardised outcome measures by Australian Physiotherapists in private practice and determined that 30% of physiotherapists used outcome measures which significantly increased to 66% after practitioner education. The authors concluded that the increase in PROM utilisation was “likely influenced by active education initiatives, professional support, and peak body position statements” [9].

### Patient reported outcome measures (PROMs)

PROMs are defined as any report of the status of a patient’s health condition that comes directly from the patient without interpretation by a clinician, their utilization within other healthcare disciplines such as Medicine, Nursing and Physiotherapy is documented [10].

Outcome measures in health care have been documented since the 1980’s [11], however, since their introduction there has only been limited research regarding their selection and implementation. More commonly research has focused on the validity, reliability, sensitivity and responsiveness of PROMs [12].

Originally many PROMs were designed as epidemiological instruments with the intention of identifying patterns of symptomatology or wellbeing, however they have evolved as tools used as pre/post measures to evaluate the influence of interventions within research studies [13]. PROMs have assisted patient-centred care to emerge as a primary approach to health care in Australia [14]. A patient centred approach allows a patient’s preference to be acknowledged and promotes shared decision making between a health-care provider and patient. Patient centred care can lead to “improvements in the quality of health systems, clinical safety and self-management by patients” [15]. The patient centred approach promotes the partnerships in health care between patients and healthcare professionals and aims to move beyond the “traditional paternalistic” approach to health care [14].

In Australia, PROMs are an emerging method of assessing health care quality. In mid-2016, The Australian Health Services Research Institute (AHSRI), investigated PROMs-related research and the application of PROMs within health services. The aim was to “describe the current status of the collection and use of PROMs in Australian health care” [16]. This study revealed that “although many organisations in the healthcare sector are interested in PROMs, their actual development, collection and use is currently patchy and inconsistent” [16]. Additionally there has been no Chiropractic specific research conducted on the use of PROMs for LBP in clinical practice.

The aim of this study was to determine the current utilisation of PROMs for LBP amongst chiropractors in Australia and to identify the potential barriers and facilitators for using PROMs for LBP in clinical practice.

## Methods

### Study design

Survey methodology was used to achieve the objectives of this research. An online cross-sectional study, was administered via the Survey Monkey™ software. The survey was purposely designed to encourage participation and increase the response rate by being succinct, convenient, user friendly and avoided the use of Chiropractic Jargon such as treatment specific terminology. The online study design allowed convenience for both the respondents and the researchers [17].

### Survey design

A cover letter to participants outlining the aim of the study and gaining consent was included to increase the completion rate of the survey. Anonymity was assured, to also increase response rate [18].

The survey design ensured each question provided relevant data to achieve the study research aims.

The survey items were designed to gain information pertaining to the aims of the study and included; current use of LBP PROMs, type and frequency used in clinical practice and the identification of potential barriers and facilitators to using PROMs for LBP in Chiropractic practice [19].

The item design was based upon the principles of Kronsik and Presser, [20] and used simple, familiar words, avoided double-barrelled questions, did not use single or double negatives, used simple syntax and avoided words with ambiguous meanings. Additionally they avoided leading or loaded questions. The design offered a range of different response modes including; multiple choice, short answer, open text box response and a five point Likert scale. Kronsik and Presser, [20] recommended that response options are clear, exhaustive and mutually exclusive. The framing of the question was intentionally neutral, in order to avoid leading the responder and minimising bias [21].

Prior to distribution, the survey instrument was pilot tested via an email invitation to a sample of convenience of chiropractors (10) in order to increase its validity, and ensure readability [22]. The findings from the pilot test revealed some terminology clarification was required in the pilot draft of the instrument and was modified prior to national distribution. The survey consisted of a total of 25 items and the electronic design of the survey was in a matrix which allowed it to be tailored to the participant’s responses, directing them towards only the items which were applicable.

The respondents who answered ‘no’ when asked if they used PROMs were redirected to a set of three items to obtain the reasons why they didn’t use them. These statements were: ‘We would like to know the reasons why you do not use PROMs in your clinic?’ and ‘Are there any ways in which you could see them being influential?’ Additionally survey respondents we asked ‘What would assist you to consider implementing PROMs in your clinic?’

The participants were issued reminder emails throughout the data collection period between June and August 2016 to encourage participation [23]. “The Dillman total design survey method” encouraged the use of follow up reminders and the suggested reminder schedule was used as a guide [24]. Reminders were sent out to the CAA members at week 2, 5, 8 and 10. CA members received reminders at week 3, 7 and 9. The reminders were sent out on alternate weeks as some participants may have been members of both CAA and CA, in order to increase the exposure to the survey and not have the participant receive two invite emails on the same day so they did not consider the approach as electronic spamming. The link to the survey was registered to the respondents email address which expired after the survey was completed, thus preventing participants completing the survey more than once.

### Survey distribution

The sample population consisted of all chiropractors registered and practicing in Australia who were members of the Chiropractic Association of Australia (CAA) and Chiropractic Australia (CA). The participants were recruited via email invitations, which included a consent form sent out by the administrators of CAA and CA organisations. A total of 3014 CAA members and 930 CA members were invited to participate totaling 3944 participants. At the time of the survey being administered, there were 4875 registered and practicing chiropractors in Australia, thus 81% of the Chiropractic profession in Australia were invited to participate in the survey.

### Data analysis

The raw survey data was tabulated and cleaned prior to the data analysis. Descriptive statistics in the form of frequency analysis were utilised which aligned with the primary objectives. The survey data was analysed by gender, age, years in practice and type of practice to gain a demographic picture of the respondents.

Descriptive statistical analysis was performed using Microsoft Excel 2013. All data were held confidentially.

### Ethics

Ethics approval for this study was granted by the human ethics committee of XXXX University. Survey participants consented that their data could be used for research purposes.

## Results

A total of 558 participants responded to the survey invitation representing 14.1% response rate. The results will be discussed in relation to; demographic information, LBP PROM utilisation, methods of PROM administration and barriers practitioners identified concerning LBP PROM utilisation.

### Demographics

Male respondents accounted for 67.6% with women representing 32.4%. The most common age range of the respondents was 35–39 years (17.2%) and the least common age range being 20–24 years of age (2%).

**Fig. 1:**
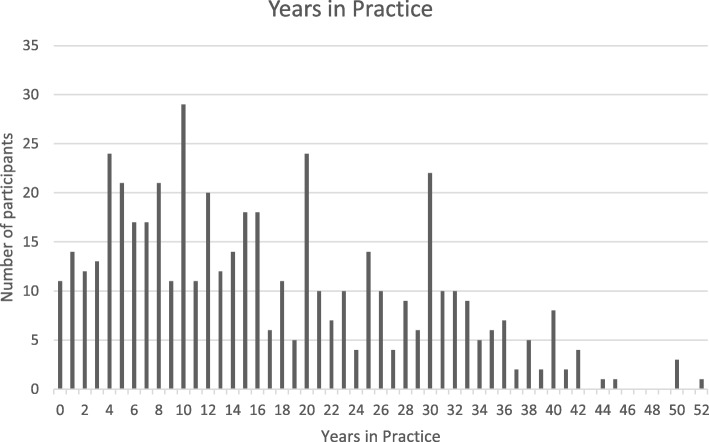
Years in practice

Practice owners or principle chiropractors accounted for 76.5% of the respondents, with the remaining 23.5% being associates. The respondent’s number of years in practice ranged between a new graduate in their first year of practice to 52 years, (Fig. 1).

### Utilisation

Of the 558 survey respondents 72.5% used PROMs in their clinical practices. The most common response for not using PROMs in clinical practice was that practitioners lacked an understanding of what PROMs really were. Other reasons stated by participants included; “Time consuming/time required to complete them, lack of knowledge about how to use them, unaware of the best ones to use and that they use verbal feedback rather than paper forms”.

### LBP PROMs specific to pain, function and health

PROMs can be designed to collect particular information from a patient, this survey examined the utilisation of LBP PROMs specific to Pain, Function and Health.

### Pain PROMs

The findings from this survey found that the three most commonly used pain PROMs for LBP on Initial consultations were the Pain diagram, Visual Analogue Scale (VAS) and Numeric Rating Scale (NRS) see Table 1. Pain diagrams were used on initial consults by 72% of the respondents, VAS used by 58% and the NRS was used by 42%. The McGill pain questionnaire (12%) was the least frequently nominated LBP PROM on initial consultations.

**Table 1:** Pain PROMs for LBP and their frequency

PROM	Freq. (n)	Initial consult	Each visit	3–6 visits	6–9 visits	9–12 visits	Weekly	Monthly	Annually	Never
LBP PAIN PROM										
Pain diagram		212	9	13	8	15	2	2	10	58
Visual analogue Scale (VAS)		167	53	45	14	36	3	10	10	43
Numeric rating scale (NRS)		119	65	45	11	26	8	8	6	74
McGill pain questionnaire		32	0	6	3	6	1	12	10	189
“Other”	31									

The most commonly used pain PROM for LBP used on each visit (non-initial) was the NRS (23%), VAS (18%) and pain diagram (3%), with NRS also the most commonly used on all subsequent visits. The three most common “other” Pain PROMs for LBP that the survey respondents noted include Functional Rating Index (FRI) (10%), Patient reported functional scale and Patient Specific Functional Scale (PSFS) (5%). Additionally some chiropractors noted that they created their own PROMs.

### Functional PROMs

The most common functional LBP PROM used on initial consultation were the Oswestry Disability Index (ODI) (38%), Functional Rating Scale (FRI) (22%), Roland Morris Questionnaire (RMQ) (10%), Fear avoidance (9%), Quebec (5%) and Bournemouth Questionnaire (5%), see Table 2.

**Table 2:** Functional PROMs for LBP and their frequency

Functional LBP PROM	Freq. (n)	Initial consult	Each visit	3–6 visits	6–9 visits	9–12 visits	Weekly	Monthly	Annually	Never
Oswestry Disability Index (ODI)		109	3	20	21	29	3	30	17	109
Roland Morris Questionnaire (RMQ)		27	2	5	8	8	1	7	8	203
Quebec Questionnaire		14	1	2	2	8	2	6	3	214
Functional Rating Index (FRI)		59	7	16	15	20	1	7	9	179
Bournemouth		14	0	5	2	7	0	1	2	223
Fear Avoidance Questionnaire		23	1	10	5	6	2	6	9	201
“Other”	28									

On each subsequent visit the most commonly use function PROM for LBP reported in the survey was FRI (2.6%), ODI (1%) and Roland Morris (.8%).

The most common “Other” functional PROM reported by chiropractors was PROMs they had created themselves.

### Health PROMs

This survey indicated that the Health Status Questionnaire (HSQ) (38%) was by far the most commonly used health PROM on initial consults and on all follow up visits, monthly and annually, see Table 3. Less commonly the RAND 36 (7.7%) and RAND 12 (7.1%) surveys were used on initial consults and follow up visits. “Other” Health Questionnaires, chiropractors reported they used were; Wellness indexes (10%), Global wellbeing scale (10%) and chiropractors creating their own Questionnaires (7%).

**Table 3:** General health PROMs and their frequency

PROM options	Freq. (n)	Initial consult	3–6 visits	6–9 visits	9–12 visits	Weekly	Monthly	Annually	Never
Health status Questionnaire		104	10	3	22	0	7	14	147
RAND SF (36)		20	2	1	4	0	10	7	222
RAND SF (12)		18	1	1	2	0	6	6	224
Dartmouth COOP charts		2	1	0	1	0	3	0	241
“Other”	19								

### Administration

The responses from this survey found that the most common method of administering PROMs in Chiropractic clinical practices in Australia was via the chiropractor and in paper form see Table 4. This method was used by 47% of the respondents. Paper based survey administered by other staff accounted for 39% of responses. Electronic/Online administration within a private practice accounted for 5.9% of responses. In paper form completed outside the clinic setting, was the preferred method by 2.9% of respondents followed by electronically/Online sent to the patient outside of the clinic setting (2.3%). “Other” methods of PROM administration included; as part of SOAP (Subjective, Objective, Assessment, and Plan) notes (42%), verbally (14%) and “online” (14%).

**Table 4:** Most common LBP PROM administration method

Response	Percentage (%)
In paper form within the clinic, administered by the Chiropractor	47.4
In paper form within the clinic, administered by other staff	39.5
Electronically/Online within the private practice	5.9
In paper form sent out to the patient in paper form outside the clinic setting	2.9
Electronically/Online sent to the patient outside the clinic setting	2.3
Other	2.0

### LBP PROMs in clinical practice

Of the survey respondents the most common purpose chiropractors in Australia identified using PROMs in clinical practice was to monitor/track progress of a patient (32%). The second most common reason was for legal requirements (19%). Specifically noted were WorkCover, third party insurance companies and the Australian Health Practitioner’s Registration Agency (AHPRA)/Chiropractic board requirements. Respondents also noted that they used LBP PROMs to gain additional information from a patient regarding their health status (9%). Confirming the effectiveness of a treatment choice (8%), patient education (4%) and creating a baseline for care (4%) were also amongst the most common reasons for using PROMs.

Of the survey respondents who used LBP PROMs, 87.2% noted that it was somewhat important or very important for a practitioner to use PROMs in clinical practice, see Table 5. Of the responses for this item 6.1% of the respondents noted that PROMs was unimportant or very unimportant for a practitioner.

**Table 5:** ‘In your clinic how important do you think PROMs are for the Practitioner?’

Answer	Very important	Somewhat important	Neutral	Unimportant	Very unimportant
Frequency	43.6%	43.6%	9.5%	3.1%	3%

The most commonly reported reasons for this included; “Only important to insurance companies, won’t change the practitioners response to treatment, waste of time, unreliable, patients who are in pain are unlikely to complete PROMs and a lack of patient literacy”.

With regards to the importance of PROMs from a patients perspective 76.6% of survey respondents stated that they felt that PROMs were important for the patient. 15.4% thought that the importance of PROMs from a patient’s perspective was neutral, leaving 8% of respondents believing PROMs were unimportant/very unimportant for patients.

The most common reason why respondents felt PROMs were important for the patient included; “so that the patient can monitor their progress and see the benefit/outcome/change/improvement”. Additionally, PROMs assist to create a background/history/reference point and ensure treatment appropriateness/efficacy. Finally patient education was also a common reason for why PROMs were important to the patient.

Respondents reasons why LBP PROMs were unimportant in order of most commonly reported included; “Patients don’t care, patients place low value on the results, patients will self-report improvements, too much paperwork for patients, too time consuming, patients already know how they feel, focuses patients attention on pain/symptoms, good communication decreases the need for PROMs, patients are sceptical of their relevance, short term patients don’t need them, the interest from insurance companies and do not relate to improvement”.

Of the survey respondents, 78.5% of the respondents indicated that PROMs were very influential or somewhat influential in their treatment plans, see Table 6. 11.2% of the respondents noted ‘neutral’ feelings over the amount of influence PROMs have within treatment plans, with 10.3% noting that PROMs were of little influence or no influence when considering treatment plans.

**Table 6:** The influence of PROMs on practitioner’s treatment plan

Answer Options	Response %
Very influential	21.1%
Somewhat influential	57.4%
Neutral	11.2%
Little influence	7.3%
No influence	3.0%

Dictating treatment choices/technique/protocols/appropriateness were the most common purpose why PROMs were identified as influential in treatment plans. The next most common reasons were to monitor patient recovery/progress, to use as a clinical guide, assist to dictate treatment timings/scheduling and to monitor the treatment success/effectiveness. The most commonly stated advantage that chiropractors in Australia noted with using PROMs in clinical practice was to monitor progress. The next most common reasons identified was to “provide objectivity, to gain additional information/data, compliance to third parties such as department of veterans’ affairs (DVA), WorkCover, AHPRA and to provide an evidence based treatment approach”.

### Barriers

Of the 558 responses 27.5% stated that they do not use PROMs in clinical practice.

The most common disadvantages/inconveniences noted that prevented practitioners using PROMs in clinical practice was the time to apply or reassess PROMs (60%). Participants stated the most common barriers when using PROMs; Patient’s reluctance to fill in forms (17%), no disadvantage (9%), the increase practitioner work load (7%) and admin workload to administer and to score the PROMs (7%).

Respondents who do not use PROMs were asked “What would assist you to consider implementing PROMs in your clinic”. The most common response was “Understanding of what specific PROMs to use and why they should be used”. Additionally, survey respondents stated that “making PROMs easier to use, having a structured system of implementation, using electronic forms, making PROM usage a compulsory requirement, offering courses/training in PROMs and nothing”, would assist in implementing PROMs.

Of the participants who did not use LBP PROMs the most common responses regarding the ways they could see PROMs being influential in clinical practice included; “Need to make a living and to do what patient wants, egos, patients regular subjective reporting is more beneficial and for marketing purposes”.

### Facilitators

The most common advice that the participants of the survey would offer to other practitioners to implement PROMs was simply to “do it” (18%), see Table 7. It was also suggested that starting with basic forms (11%), a simple administration system (10%), using electronic forms (8%) and being consistent with the implementation (7%) would assist other practitioners. Training reception staff to administer (6%) the PROMs is also advice that the survey participants stated may assist others to implement PROMs in their clinics.

**Table 7:** Facilitators

Response	Percentage
Encourage other Practitioners to implement PROMs (Do it) [Practitioners feel that other practitioners should be using them and want to encourage more use]	18
Start with basic forms	11
Have a simple administration system.	10
Availability online/electronically or sent electronical through clinic software	8
Be consistent when using them	7
Train reception staff to administer them	6

## Discussion

This research was intended to determine the extent of the utilisation of standardised LBP PROMs amongst the Chiropractic workforce in Australia and to identify barriers and facilitators to their use.

### Demographics

Basic demographic data was collected, therefore any assumptions about the chiropractors, type of practice, techniques used or chiropractic philosophy, cannot be made. The survey was intentionally designed to be short in order to increase participation, however this limited the amount of information collected. The methodology for this research is a descriptive observational study, designed to observe the sample population without interference [25].

The survey revealed that 72.5% of respondents used PROMs in their Chiropractic clinical practices. The 27.5% of respondents who reported they do not use PROMs were redirected to a set of three items to obtain the reasons why they don’t use them in clinical practice.

It was important to discover why LBP PROMs were not being used by practitioners. The opinions of the practitioners that don’t use them provided information regarding barriers to their use. The survey uncovered the fact that practitioners don’t understand what specific PROMs should be used, how and when. They stated that by making PROMs easier to use and having a structured process for clinical implementation may assist. Also of importance is that respondents stated that offering training/courses to educate practitioners about PROM use and implementation would assist them to consider implementing PROMs.

Of the 558 participants who completed the survey 67.6% of them were men. The AHPRA collects statistics regarding the Chiropractic population in Australia. During the data collection period of this survey the AHPRA statistics were accessed and compared to the demographics of this survey. AHPRA statistics in September 2016, report that there were more male practicing chiropractors than females (Male- 3024 (61.5%), Female- 1864 (38.5%) [26] this may be the reason for more male participants than female participants in this survey. The most common age ranges of the participants in this study were 35–39, 25–29, 30–34 and 40–44 years. These survey findings were consistent with the most populated age brackets of practicing chiropractors according to AHPRA, 2016, statistics [26].

Practice owners or principle chiropractors accounted for 76.5% of the respondents. This information was something that has not previously been reported on within Australia and there is no data to use as a comparison. This information may be of interest as being a practice owner/Principle Chiropractor may dictate the clinical procedures with regarding to utilising PROMs. Conversely an associate Chiropractor may not have the ability to use PROMs due to clinical procedure beyond their control. However these items were not asked in the survey and therefore can not be assumed.

The respondent’s number of years in practice varied between a new graduate in their first year of practice to 52 years, with the most common amount of years in practice being 10 years. The findings from the Australian Chiropractic Research Network (ACORN) workforce article discussed that “the average number of years in practice was 15.8 (SD = 11.3) years”, which is very similar to the findings of this research [27].

The most commonly stated method of administering PROMs in Chiropractic clinical practices in Australia was in paper form, within the clinic, and administered by the Chiropractor (47.4%) Although it would be suspected that electronic versions of PROMs would be highly utilised due to the low cost and high accessibility, traditional paper and pencil format is still much more commonly used in clinical settings [28]. Even though PROMs are most often used in paper form, research indicates that the use of electronic PROMs may be more effective than paper based PROMs, and thus the need for more development in this area [29].

### Utilisation

PROMs are intrinsically related to providing quality health care and have become more readily utilised in clinical practice and since the early 1990’s they were primarily used to; increase knowledge of the natural history of a condition, evaluate treatment efficacy and assess the quality of care [30].

The most common reason chiropractors in Australia identified for using PROMs was to monitor and track progress of a patient. As stated by Yeoman & Leibensen, [6], when used appropriately PROMs effectively measure a change in health status in relation to pain management and physical impairment.

A large portion of the respondents to the survey reported that they use PROMs due to legal requirements implied by work cover, third party insurance companies and AHPRA/Chiropractic board requirements.

Work cover requirements for chiropractors vary slightly between the states and territories of Australia. However, all states and territories indicate that it is “expected that all healthcare professionals providing services to injured workers will routinely use outcome measures every 4–6 weeks to clinically justify their treatment. To capture early recovery, it is important measurement commences as early as possible” [31]. The Victorian government website provides health care providers with an extensive list of PROMs divided into condition specific sections to assist practitioners in selecting the recommended PROM. Although the work cover policy states that it is expected health care practitioners use PROMs, it is not mandatory. There does not appear to be any specific guidelines that are implied by private health fund companies in Australia with regards to the use of PROMs.

Practitioners assuming that it is mandatory to use PROMs, may be due to poor communication between work cover/private health companies and practicing chiropractors.

The Chiropractic Board of Australia code of conduct policy recommends that a patient review/reassessment should, but not must, include validated objective and subjective outcome measures.

Other reasons respondents noted for PROM use included; to confirm the effectiveness of a treatment choice, educating patients and creating a baseline for care. These views within the Australia Chiropractic workforce are echoed amongst other health care professions. The findings are consistent with the study by Kyte, Calvert, Van der Wees, Hove, Tolan & Hill, [32] “An introduction to patient-reported outcome measures (PROMs) in Physiotherapy” where they reported that PROMs assisted Physiotherapists in their clinical decision making process for diagnosing and applying treatment to patients. The routine use of PROMs in clinical practice may allow health care practitioners to track treatment outcomes which would assist in designing effective management strategies [32]. Holmes et al. [30], confirmed that the information collected from PROMs can provide baseline data regarding a patients’ current health status and be used to predict change and used when setting goals.

PROMs have been shown to be a valuable clinical tool as they increase a patients’ understanding of their condition and assist the patient to monitor their recovery and progress over time [33]. It has also been seen that the use of PROMs are effective in educating patients in relation to their condition which enables them to cope better with their illness [34].

### Pain PROMs

A patient’s subjective complaint of pain can be extremely difficult to quantify, yet it is seen as the most important criterion when monitoring change in a patient’s condition [12]. The most accurate measurement of a patient’s pain level comes directly from their own description [35]. The three most commonly used pain PROM for LBP on initial consults reported by the surveyed population were Pain diagram, Visual Analogue Scale (VAS) and Numeric Rating Scale (NRS).

#### Pain diagrams

Pain diagrams generally consist of a diagram outline of the human body from the front and from the back. The intention is for the patient to identify the location and quality of pain by drawing and using symbols on the diagram. Quantifying pain type, location and distribution are important factors for clinicians managing patients with musculoskeletal disorders, such as LBP. Measuring pain can serve as an outcome measure which can be useful when determining prognostic factors [36]. Pain diagrams are valuable as they can determine pain arising from a range of conditions. Pain distribution and pain location can be reliably and consistently measured on body pain diagrams [36]. This current study indicated that chiropractors in Australia use pain diagrams as their most common form of pain assessment when in clinical practice.

#### Visual Analogue Scale (VAS)

VAS is a psychometric instrument which measures subjective characteristics or attitudes regarding a patient’s pain. A VAS is usually a horizontal line, 10 cm in length, with descriptors at each end, e.g. no pain and extreme pain [37]. Reliability and validity of the VAS has been widely reported in research; it is often used in epidemiologic and clinical research to measure the intensity or frequency of patient’s symptoms [38]. The VAS is effective in its ability to detect change in patients with chronic inflammatory or degenerative joint pain [39]. It is commonly accepted that chiropractors treat patients with chronic inflammatory and degenerative joint disease and therefore is a sound choice for chiropractors. This survey revealed that the VAS was a popular choice amongst the survey respondents.

#### Numeric Rating Scale (NRS)

A scale consisting of 11 numbers (0–10), where the patient is instructed to circle the most appropriate number which represents their pain level. NRS are a popular clinical assessment tool due to their ease of administration and simplicity to complete [40].

In a study by Ferreira-Valente, Pais-Ribeiro and Jensen, [41] it was shown that the NRS is more sensitive and responsive than the VAS. Additionally, the NRS was the preferred PROM by patients and clinicians due to its simplistic nature and ease of administration. The NRS is a common LBP PROM in Chiropractic literature [7]. The literature supports the NRS as both a reliable and valid method of measuring pain intensity across many populations [41].

### Functional LBP PROMs

In recent decades, numerous scoring systems have been developed to assess the functional status of patients with LBP [42]. Functional PROMs are instruments designed to evaluate the functional capacity of a patient [43]. This survey revealed the most common functional LBP PROMs used on initial consultations reported by respondents to be the ODI, FRI and RMQ.

These findings are consistent with a large scale postal questionnaire, distributed in 1998 to 581 rehabilitation centres throughout Europe, which reported the ODI and the RDI were the most frequently reported PROMs [44].

#### Oswestry Disability Index (ODI)

“The ODI is divided into ten sections to assess the level of pain and interference with several physical activities including; sleeping, self-care, sex life, social life and travelling” [45]. Each individual item has six possible responses scored 0–5. The score is added and yields a percentage of disability that the patient is experiencing. Clement et al., [46] cited that the ODI was the most commonly used PROM relating to function in patients with low back conditions, with RMQ being the second most common. ODI and RMQ have both been proven to be reliable, responsive and valid, the ODI has been the most extensively researched PROM and provides superior clinical interpretability [47].

#### Functional Rating Index (FRI)

The FRI was developed as an assessment tool to quantify a patient’s current level of pain and dysfunction in a reliable and valid manner in relation to their LBP [48]. The FRI is a 10-item scoring instrument designed to measure the patient’s perception of their function with regards to activities of daily living (ADLs), pain intensity and frequency. Results indicate that the higher the score the higher the perception of dysfunction and pain [42]. The survey responses from the Australian Chiropractic workforce indicated that the FRI was the second most common functional LBP PROM used on initial consultations.

#### Roland Morris Questionnaire (RMQ)

The RMQ is a commonly utilised instrument used to measure spinal disability [12]. It is made up of 24 statements about patient’s ADL’s and their limitations due to their LBP [45]. The RMQ has been proven to be effective when specifically measuring a patient’s function. The RMQ is the superior instrument in terms of its clinometric performance when comparing other back pain specific instruments as it is reliable and responsive [49]. The research completed by Hinton et al., [8] investigating the outcome measures and their everyday use in Chiropractic practice, had similar findings to this study, in their survey of American chiropractors, reporting that RMQ is frequently used by chiropractors in clinical practice.

### Health PROMs

Health PROMs gather information regarding a patient’s general health status. They are clinically beneficial as they collect information that is not condition specific and therefore can be applied to virtually any condition [12]. The application of general health questionnaires is vast, as they can be applied to any patient, with any condition, seeking treatment from any discipline. Reduced quality of life perception has been noted as an important symptom, and can be correlated with a patient’s physical disability [50]. This study concluded that health status questionnaire (HSQ) was by far the most commonly used health PROM. Less common were the RAND 36 and RAND 12 which was used on initial consultations and follow up visits.

#### Health status questionnaire

The HSQ is an instrument designed to enable measurement of a patient’s reported well-being. It is a 39-item instrument that assesses the patient’s physical and mental health status. This instrument includes 1–5 Likert scale response choices and asks the patient to circle the appropriate option. The lower the score the more disability or decreased quality of life the patient is experiencing. Literature indicates that the HSQ is a valid and sensitive instrument to distinguishing medical and psychiatric conditions [50]. This research concluded that the HSQ was by far the most common Health PROM used within Chiropractic clinics in Australia.

#### SF-36 and 12

Initially developed in the 1980’s are the well-used health questionnaires. They are designed to measure eight key health attributes; including patient’s physical, social and mental well-being with each attribute scored using a Likert scale. Health questionnaires have been modified over time due to initial low completion rates of respondent’s to the “long” versions of the questionnaires. In 1986 a shorter version, Short form (SF20) of 20 health questions was designed, now available in an even shorter version (SF12). In shortening the instrument researchers and practitioners noted that there is trade-off between practicality and validity [12].

Hinton et al., [8] noted that only 8% of respondents use the SF 36/12 health status questionnaire. However, within this research project SF36 and SF12 were noted as common choices for health PROMs among chiropractors.

### Barriers

A key aim of this research was to determine the barriers chiropractors in Australia report that prevents LBP PROMs utilisation in Clinical practice. The most common reasons that chiropractors do not use PROMs was that they are unaware of what PROMs really are, lack of knowledge about how to use them, are unsure of the best ones to use and additionally that they are time consuming, see Table 8.

**Table 8:** Barriers clinicians face in clinical practice

Response	Percentage
Not sure what PROMs really are/ Lack of knowledge	18.6%
Time consuming/required	17.4%
Unsure about how to use them	9.3%
Unaware of the best ones to use	8.1%
Use verbal feedback rather than paper forms	4.6%

Holmes et al., [30], commented that “clinician knowledge and education, organisation support, selection and application of PROMs” were all barriers clinicians face. This issue may be a global issue as de Jong et al., [51] reported that clinicians have concern over which PROM to use and when, which may severely limit potential positive benefits. A lack of training and education regarding PROM use has been seen as a barrier to PROMs use [52]. These findings are mirrored in a systematic review of allied health professionals, which reported a number of barriers that prevent routine outcome measurement usage, these include: “the absence of effective PROM-specific organisational and peer-support; and a lack of knowledge and confidence about using outcome measures” [53]. In a survey of Australian physiotherapist five barriers to using outcome measures were identified; time required to administer the tests and lack of familiarity with functional tests were nominated by more than 80% of respondents; these findings are similar to the findings of this study [9].

The survey participants were asked “What disadvantages/inconveniences do you see with using PROMs in your clinic?” The most common response was that the participants stated that the time to apply or reassess PROMs. Clinicians may experience time constraints working in private practices which is seen to be a factor in practitioners considering using them. The process involved with using PROMs may imply additional administrative burden, particularly if there aren’t structure support systems in place from a resource perspective [54].

Whilst many clinicians support the concept of using PROMs to aid in monitoring progress and enhancing communication, there are concerns about the time and support necessary for successful implementation [13].

When the respondents were asked to provide advice to other practitioners to assist in implement PROMs in their clinics, the most common response was to encourage other practitioners, to “do it”. Survey respondents also suggested that starting with basic forms, a simple administration system, using electronic forms and being consistent with the implementation would assist other practitioners. These findings were also seen in Wagel, [55] agreeing that simple administration systems or making the PROM process simple, is the best way to implement PROMs.

Training reception staff to administer the PROMs is also advice that the survey participants reported that may assist others to implement PROMs into their clinics. It is important to have an efficient PROM collection system and well trained staff involved in implementing the process [32].

An understanding of what specific PROMs are used and why to use them was the most common idea to assist PROM implementation. Additionally respondents stated that making PROMs easier to use, having a structured system of implementation, using electronic forms, making PROM usage a compulsory requirement or offering courses/training in PROMs. As discussed above simple, effective support systems within the clinical process would assist PROM implementation.

Due to the low response rate these survey findings can not be generalised throughout the Chiropractic profession of Australia, however this research has established the need to educate chiropractors in Australia regarding LBP PROMs to improve knowledge and potential utilisation within clinical practice. Future studies investigating the use of PROMs through rigorously developed surveys returning high response rates, would allow additional data to be collected regarding PROM use amongst the practicing Chiropractic population.

### Limitations

The low response rate for the survey participation may be seen as a limitation of this study. Despite web-based surveys being a common method of information collection, there is debate regarding the success and usefulness particularly amongst health care professionals. Findings from Cunningham et al., [56] indicate that health care professionals are often a group with low survey response rates [56]. There has been a steady downward trend in lower response rates to surveys, particularly amongst clinicians. Factors such as an accurate mailing list, relevance of the topic to the participant, endorsement and multiple mailings of the survey have shown to increase response rates [57, 58].

Attempts were made by the authors to minimise all biases, however bias is a pervasive problem in the design of surveys/questionnaires [59]. The authors aimed to minimise selection bias by inviting the majority of the Chiropractic profession in Australia to participate. This may however reflect an unintentional responder bias in that while 3944 chiropractors were eligible, only 558 completed the survey. The participants were recruited through CAA and CA, which are independent organisations. Using CAA and CA members as the participants may have potentially create a population bias as the responders to the survey may not be representative of the entire Chiropractic workforce in Australia. The number of respondents that skipped items can also be seen as a limitation of the study.

Additionally, selection bias can occur between the participants and non-participants thus creating an unintentional bias. Therefore, a low response rate may also increase the possibility of a non-responder bias issue.

The responder bias may have been amplified due to the low response rate. It is possible that Chiropractors who do not use PROMs may have been less likely to participate in the survey, which voided the ability to statistically analyse study sub groups within the survey population. It is still possible that non-respondents may have different attitudes and may have responded to the questions differently than the respondents. Additionally offering incentives such as gift vouchers, cash or CPD points may have helped to motivate survey participation and increase response rates [60]. However in this survey, ensuring the survey participants remained anonymous was considered vital to gain accurate and honest responses and therefore these incentives were not used.

The methodology of the survey could have been strengthened with focus groups, face validity testing, readability scales and further pilot testing, however, given this was a preliminary study to determine PROM use amongst Chiropractors in Australia they were not carried out and is a further limitation of the study.

## Conclusion

This research project aimed to determine the current utilisation of PROMs for LBP amongst chiropractors in Australia, and this was achieved through a national survey of 3944 chiropractors. The survey found that 72.5% of respondents use LBP PROMs in clinical practice. The survey findings determined the three most commonly used pain PROMs for LBP were Pain diagram, VAS and NRS. The most common functional LBP PROMs used on initial consultation were the Oswestry, FRI and RMQ. The Health Status Questionnaire (HSQ) was by far the most frequently used health LBP PROM, followed by the RAND Health Questionnaires. The commonly used LBP PROMs amongst chiropractors in Australia varies slightly from the most commonly used LBP PROMs seen within Chiropractic literature. The most common barrier chiropractors identified that prevent LBP PROM use is that they are not sure what PROMs really are, how to use them, and that they are time consuming. Facilitating factors to implement PROMs include using simple administration systems, utilising electronic forms and consistency. The findings from the survey suggests there is significant PROM usage within the Australian Chiropractic profession although due to the relatively low response rate this can not at this stage be generalised to the profession as a whole. Given these findings practitioner education and the creation of systematic guidelines to assist LBP PROM implementation is recommended.

## Abbreviations

ABS: Australian Bureau of Statistics
ACORN: The Australian Chiropractic Research Network
ADL’s: Activities of daily living
AHPRA: Australian health practitioner’s registration agency
AHSRI: Australian Health Services Research Institute
CA: Chiropractic Australia
CAA: Chiropractic Association of Australia
CQU: Central Queensland University
DVA: Department of veterans’ affairs
FRI: Functional Rating Index
HSQ: Health Status Questionnaire
LBP: Low back pain
NHS: The National Health Service (UK)
NRS: Numeric Rating Scale
ODI: Oswestry Disability Index
PROM: Patient reported outcome measure
PROMs: Patient reported outcome measures
PSFS: Patient Specific Functional Scale
SF12: Short form 12
SF20: Short form 20
SF36: Short form 36
SOAP: Subjective, Objective, Assessment, and Plan
VAS: Visual Analogue Scale
